# Thiamidol containing treatment regimens in facial hyperpigmentation: An international multi‐centre approach consisting of a double‐blind, controlled, split‐face study and of an open‐label, real‐world study

**DOI:** 10.1111/ics.12626

**Published:** 2020-07-20

**Authors:** W. G. Philipp‐Dormston, A. Vila Echagüe, S. H. Pérez Damonte, J. Riedel, A. Filbry, K. Warnke, C. Lofrano, D. Roggenkamp, G. Nippel

**Affiliations:** ^1^ Hautzentrum Köln Schillingsrotter Str. 39‐41 Köln 50996 Germany; ^2^ Faculty of Health University Witten/Herdecke Witten 58455 Germany; ^3^ Centro de Referencia en Tratamiento Laser Av. Del Libertador 662, Piso 17, depto. 42 Buenos Aires Argentina; ^4^ CLAIM José Bonifacio 717 Buenos Aires Argentina; ^5^ BDF Argentina Av Triunvirato 2902 Buenos Aires C1427 AAP CABA Argentina; ^6^ Beiersdorf AG Unnastrasse 48 Hamburg 20245 Germany

**Keywords:** claim substantiation, emulsions, hyperpigmentation, skin physiology, structure, Thiamidol

## Abstract

**Objective:**

Tyrosinase is the rate‐limiting enzyme in melanogenesis. Thiamidol is the most potent inhibitor of human tyrosinase out of 50 000 tested compounds. In clinical studies, it was shown to improve facial hyperpigmentation, post‐inflammatory hyperpigmentation and age spots significantly. To identify the optimal number of daily Thiamidol applications, we conducted a split‐face study comparing the efficacy and tolerability of four‐times with two‐times daily application. Subsequently, we evaluated the efficacy and tolerability of a typical face care regimen containing Thiamidol in a real‐world study.

**Methods:**

The split‐face study was double‐blind, randomized, controlled, including two Thiamidol containing products (serum and day care SPF 30). The serum was applied twice daily on one half of the face and the day care SPF30 twice‐daily on the whole face. The real‐world study was open‐label, observational, including three Thiamidol containing products (day care SPF 30 in the morning, serum and night care in the evening). In both studies, subjects with mild‐to‐moderate facial hyperpigmentation applied the products over 12 weeks. Assessments included clinical and subjective grading of hyperpigmentation, skin condition, hemi‐/modified MASI, chromameter and clinical photography.

**Results:**

In the split‐face study (*n* = 34), hyperpigmentation, skin roughness and hMASI improved all significantly (*P* < 0.001) versus baseline, with first visible results after two weeks of twice‐daily application. The four‐times daily application led to significant improvement versus the two‐times daily application. In the real‐world study (*n* = 83), all evaluated parameters, including skin condition and chromametry (*n* = 30), improved significantly (*P* < 0.001) in comparison with baseline and the corresponding preceding visits. The subjects judged the cosmetic properties of the products positively. In both studies, the products were well tolerated.

**Conclusion:**

Four‐times daily Thiamidol improves facial hyperpigmentation significantly more than two‐times daily and is well tolerated by the subjects. The real‐world study with a typical face care regimen containing Thiamidol shows improvement of facial hyperpigmentation and confirms tolerability. Furthermore, the data provide evidence for the suitability of this three‐product Thiamidol regimen for day‐to‐day life.

## Graphical abstract

Face care regimen containing Thiamidol, improves facial hyperpigmentation and is well tolerated.

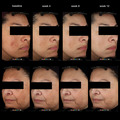

## Résumé

**Objectif:**

La tyrosinase est l’enzyme qui limite le taux de mélanogénèse. Le Thiamidol est le plus puissant inhibiteur de la tyrosinase humaine parmi les 50 000 composés testés. Lors d’études cliniques, il a été démontré qu’il améliore de manière significative l’hyperpigmentation du visage, l’hyperpigmentation post‐inflammatoire et les taches de vieillesse. Afin d’identifier le nombre optimal d’applications quotidiennes de Thiamidol, nous avons mené une étude hémi‐visage comparant l’efficacité et la tolérance d’une application quatre fois par jour à une application deux fois par jour. Par la suite, nous avons évalué l’efficacité et la tolérance d’un régime de soins du visage typique contenant du Thiamidol dans le cadre d’une étude en situation réelle.

**Méthodes:**

L’étude hémi‐visage était en double aveugle, randomisée et contrôlée, incluant deux produits contenant du Thiamidol (sérum et soin de jour SPF 30). Le sérum a été appliqué deux fois par jour sur une moitié du visage, et le soin de jour SPF30 deux fois par jour sur l’ensemble du visage. L’étude en situation réelle était ouverte, observationnelle, et comprenait trois produits contenant du Thiamidol (soin de jour SPF 30 le matin, sérum et soin de nuit le soir). Dans les deux études, les sujets présentant une hyperpigmentation faciale légère à modérée ont appliqué les produits pendant 12 semaines. Les évaluations comprenaient une évaluation clinique et subjective de l’hyperpigmentation, de l’état de la peau, du hémi/modifié MASI, du chromamètre et de la photographie clinique.

**Résultats:**

Dans l’étude hémi‐visage (n = 34), l’hyperpigmentation, la rugosité de la peau et l’hMASI se sont tous améliorées de manière significative (*P* < 0.001) par rapport à la ligne de base, avec des effets devenus visibles aprés deux semaines d’application deux fois par jour. L’application quatre fois par jour a apporté une amélioration significative par rapport à l’application deux fois par jour. Dans l’étude en situation réelle (n = 83), tous les paramѐtres évalués, y compris l’état de la peau et la chromamètrie (*n* = 30) se sont améliorés significativement (*P* < 0.001) par rapport à la ligne de base et aux visites précédentes correspondantes. Les sujets ont jugé positivement les propriétés cosmétiques des produits. Dans les deux études, les produits ont été bien tolérés.

**Conclusion:**

L’application de Thiamidol quatre fois par jour, améliore l’hyperpigmentation du visage de maniѐre plus significative que deux fois par jour et est bien toléré par les sujets. L’étude en situation réelle avec un régime de soins du visage typique contenant du Thiamidol montre une amélioration de l’hyperpigmentation faciale et confirme la tolérance. En outre, les données fournissent des preuves de l’adéquation de ce régime de trois produits à base de Thiamidol pour le soin quotidien.

## Introduction

Hyperpigmentation is a frequent cosmetic problem characterized by hyperpigmentation on sun‐exposed areas because of increased melanogenesis [1, 2]. Although the natural sun‐induced production of melanin is desirable for its photoprotective function and the tanning effect, accumulation of abnormal amounts in specific parts of the skin resulting in darker patches is undesirable and poses an aesthetic problem [3, 4, 5].

The first rate‐limiting step of melanogenesis is the oxidation of tyrosine to dopaquinone by tyrosinase [6, 7]. Therefore, tyrosinase inhibitors are in the focus of the research for the treatment of hyperpigmentation [7, 8, 9, 10]. Acting immediately and reversibly, they are considered safer than, for example hydroquinone [11] which is not allowed in the EU for cosmetic use [12]. So far, most tyrosinase inhibitors lack clinical efficacy [13] because they were identified by testing on mushroom and not on human tyrosinase [8, 14, 15]. The purification of soluble variants of human tyrosinase [16] allowed the identification of Thiamidol (isobutylamido thiazolyl resorcinol) as its most potent inhibitor [11, 17, 18] out of 50 000 screened compounds.

In melanocyte cultures, Thiamidol was superior to widely used tyrosinase inhibitors such as kojic acid, arbutin, or hydroquinone showing potent and reversible inhibition of melanin production [17]. In addition, the resorcinol derivative being a strictly competitive tyrosinase inhibitor and not a substrate is not converted to a toxic and potentially leukoderma‐inducing quinone [17]. *In vivo* Thiamidol twice‐daily reduced visibly and significantly age spots [17], the modified melasma area and severity index (mMASI) [19] in comparison with control (sunscreen only; *P* ≤ 0.001) [20], and post‐inflammatory acne hyperpigmentation [21] and was always very well tolerated.

So far, its efficacy and tolerability were shown in twice‐daily applications. To identify the optimal number of daily applications, we did a split‐face study in a clinical setting, comparing the efficacy and tolerability of four‐times with two‐times daily application in women with mild‐to‐moderate facial hyperpigmentation. In a second study in the same target group, we investigated the efficacy and tolerability in a day‐to‐day life setting (real‐world study) using a typical three‐product face care regimen [22] (serum, day care with SPF 30 and night care cream) containing Thiamidol.

## Materials and methods

### Split‐face study

#### Study design

This was a single‐centre, double‐blind, randomized, controlled, split‐face clinical study conducted in a centre in the United States (Texas Research Center, Texas 75081).

After approval by the institutional review board, the study was conducted following the Declaration of Helsinki and the ICH GCP guidelines as applicable to cosmetic products. Before study inclusion, the subjects received the patient information and provided written, informed consent conforming to Title 21 Code of Federal Regulations (CFR) 50.25, including consent for photography.

#### Study population and treatment

Eligible for the study were healthy subjects (25–65 years, Fitzpatrick skin type I–IV) with mild‐to‐moderate hyperpigmentation (3–6 according to a modified Griffith’s 10‐point scale, where 0 = none and 9 = severe [23]) on both sides of the face and willing to use the two test products. Pregnancy, allergies to skin care products, hormone replacement therapy or oral contraception within the previous 3 months, topical hyperpigmentation treatment within the previous 4 months, any topical or systemic medication known to affect skin ageing or dyschromia within the previous 8 weeks, any facial treatments, or photosensitizing or immunosuppressive drug application within the previous 6 months, high energy treatments, plastic surgery, or ablative laser resurfacing within the previous 12 months, extensive UV or other irradiation during the study, suntan, scars, birthmarks, skin cancer, chronic diseases (e.g. rosacea, psoriasis and acne) or strong hair growth on the face, poorly adjusted hypo‐ or hyperthyreosis, health condition or pre‐existing or dormant dermatologic disease on the face that could interfere with the outcome of the study, or concomitant participation to another clinical study were all exclusion criteria.

The subjects were allowed to follow their usual make‐up and cleansing routine and applied the treatment regimen for 12 weeks as follows: Twice‐daily serum on the assigned left or right side of the face and day care with SPF30 on the whole face, that means, one side of the face received four Thiamidol applications and the other two applications (Table 1); the subjects were not allowed to use any face care or make‐up product in the mornings of the study visits. The INCI compositions of the study products are described in Table 2.

**Table 1:** Overview of methods of the two studies (split‐face and real‐world)

Study	Split‐face	Real‐world	
Centre	Texas (USA)	Germany (DE)	Argentina (AR)
Design	Double‐blind, randomized, controlled, split‐face	Single‐arm, open‐label, observational, real‐world study	
Test products	Serum	Serum	
	Day care SPF30	Day care SPF30	
		Night care	
Treatment	Morning and Evening: Serum on half of the face and Day Care SPF30 on the whole face	Morning: Day Care SPF30	
		Evening: Serum and Night Care	
Visits	Baseline, Weeks 2, 4, 8, 12	Baseline, Weeks 4, 8, 12	
Assessments	Clinical grading	Clinical grading	Clinical grading
	hMASI	mMASI	–
	Clinical photography	–	Clinical photography
	–	–	Dermoprime^®^
	–	–	Chromameter
	Self‐assessment	Self‐assessment	Self‐assessment

hMASI: hemi‐modified melasma area and severity index, mMASI: modified melasma area and severity index.

**Table 2:** INCI composition of the test products

Test product	INCI list
Serum (S)	Aqua, Alcohol Denat, Butylene Glycol, Glycerin, Octocrylene,Isopropyl Palmitate, Cetearyl Isononanoate, Distarch Phosphate, Methylpropanediol, Isobutylamido Thiazolyl Resorcinol, Sodium Ascorbyl Phosphate, Sodium Hyaluronate, Glycyrrhiza Inflata RootExtract, Tocopherol, Glucosylrutin, Sodium Stearoyl Glutamate, Glyceryl Stearate, Sodium Polyacrylate, Dimethicone, Isoquercitrin, Citric Acid, Sodium Chloride, Trisodium Edta, Caprylyl Glycol, Phenoxyethanol, Parfum
Day care SPF30 (DC)	Aqua, Homosalate, Alcohol Denat, Butyl Methoxydibenzoylmethane, Ethylhexyl Salicylate, Ethylhexyl Triazone, Bis‐Ethylhexyloxyphenol, Methoxyphenyl Triazine, Butylene Glycol, Dicaprylate/Dicaprate, Tapioca Starch, Distarch Phosphate, C12‐15 Alkyl Benzoate, Phenylbenzimidazole Sulfonic Acid, Isobutylamido Thiazolyl Resorcinol, Glycyrrhiza Inflata Root Extract, Tocopherol, Glucosylrutin, Isoquercitrin, Glycerin, Cetyl Alcohol, Stearyl Alcohol, Sodium Chloride, Xanthan Gum, Carbomer, Sodium Hydroxide, Glyceryl Stearate, Sodium Stearoyl Glutamate, Dimethicone, Phenoxyethanol, Trisodium Edta, Parfum
Night care cream (NC)	Aqua, Glycerin, Isopropyl Palmitate, Alcohol Denat, Cetearyl Isononanoate, Squalane, Panthenol, Glyceryl Stearate Citrate, Cetearyl Alcohol, Hydrogenated Coco‐Glycerides, Butyrospermum Parkii Butter, Methylpropanediol, Lauroyl Lysine, Isobutylamido Thiazolyl Resorcinol, Glycyrrhiza Inflata Root Extract, Tocopherol, Glucosylrutin, Carbomer, Chondrus Crispus Extract, Sodium Hydroxide, Isoquercitrin, Trisodium Edta, Phenoxyethanol, Parfum

#### Assessments

Evaluations (Table 1) were conducted at visit 1 (baseline), visit 2 (week 2), visit 3 (week 4), visit 4 (week 8) and visit 5 (week 12) and included clinical photography [24], clinical grading of hyperpigmentation, skin roughness (baseline and week 12) and subjective assessment of hyperpigmentation (both with the modified Griffiths’ 10‐point scale [23]), and hMASI (including area [lesion size], darkness [pigment intensity] and homogeneity [19] for half of the face [score 0–24, i.e. hemi‐MASI]) (weeks 4, 8 and 12). Full‐face images were taken using a VISIA CR Photo station (Canfield Imaging Systems, Fairfield, New Jersey, USA) with a Canon Mark digital SLR camera (Canon Incorporated, Tokyo, Japan) under following lighting conditions: standard 1 and 2 and cross‐polarized. The subjects acclimated at 20–23 °C and 35–65% relative humidity, at least 15 min prior to participating in any evaluation procedures.

### Real‐world study

#### Study design

This was a single‐arm, open‐label, prospective, observational, real‐world study conducted in one centre in Germany (HAUTZENTRUM Köln) and two centres in Argentina (CLAIM, Buenos Aires; Laser Therapy Clinic Dr. Agustina Vila Echagüe, Buenos Aires). In Germany, the study received approval by the local Ethics Committee and in Argentina by the corresponding Institutional Review Board. The study was conducted according to the Declaration of Helsinki and the ICH GCP guidelines as applicable to cosmetic products. All subjects received the patient information and provided written, informed consent, including photography.

#### Study population and treatment

Healthy subjects (25–60 years, Fitzpatrick skin type I–IV) with mild‐to‐moderate facial hyperpigmentation on both sides of the face and willing to use the three‐product treatment regimen were included. Pregnancy, or hormone replacement therapy within the previous 3 months, topical hyperpigmentation treatment within the previous 2 months, any facial treatments, or photosensitizing or immunosuppressive drug application within the previous 6 months, extensive UV or other irradiation during the study, suntan, scars, birthmarks, skin cancer, chronic diseases (e.g. rosacea, psoriasis and acne) or strong hair growth on the face, poorly adjusted hypo‐ or hyperthyreosis, known allergic reactions to any of the ingredients of the test regimen, or concomitant participation to another clinical study were all exclusion criteria.

The subjects were allowed to follow their usual make‐up, cleansing, and sunscreen routine and applied the treatment regimen for 12 weeks as follows (Table 1):

- In the morning, day care cream with SPF 30;
- In the evening, serum and night care cream.

The subjects were not allowed to use any face care product in the mornings of the study visits. The INCI compositions of the study products are described in Table 2.

#### Assessments

Evaluations (Table 1) were conducted at visit 1 (baseline), visit 2 (week 4), visit 3 (week 8) and visit 4 (week 12). Subjective assessments included evenness, radiance, smoothness and hyperpigmentation all with the modified Griffiths’ 10‐point scale [23]). The clinical assessments included skin condition (evenness, radiance and smoothness on a 5‐point scale, 0 = extreme, e.g. extremely uneven skin tone, 4 = absent, e.g. very even skin tone) in both centres, mMASI (modified MASI without the homogeneity factor [19, 25]) in Germany, and chromameter [26] (Chroma Meter CR‐400^®^ Konica Minolta, Japan) and clinical photography [24] in Argentina (Table 1). Full‐face digital images (right and left side views) were taken using the Fotofinder Portrait^®^ base/UVSCAN^®^ [27] with a Canon C10 digital camera under standard lightening and the Dermoprime^®^. Before the instrumental evaluations, the subjects acclimatized for 15 min at a temperature of 20–23 °C and relative humidity of 35–65%. At the end of the study (visit 4), the subjects also answered (yes/no) a product performance questionnaire.

### Statistics

We analysed the data descriptively (number, percentage, mean, standard deviation [SD], minimum and maximum) and used the Wilcoxon matched‐pairs signed‐ranks test to compare visits and face sides. In the case of multiple comparisons, we used the Bonferroni–Holm correction of the level of significance. Differences were considered statistically significant at a *P* < 0.005 level.

## Results

### Split‐face study

#### Demographic characteristics

Between April and August 2017, a total of 34 females aged 49.5 ± 8.5 years (range 25–64) were enrolled (Table 3).

**Table 3:** Demographic characteristics of the subjects of both studies (split‐face and real‐world)

Study	Split‐face	Real‐world	
Centre (*n*)	USA (*n* = 34)	Germany (*n* = 32)	Argentina (*n* = 51)
Female/Male, *n*	34/0	31/1	51/0
Age, years			
Mean (SD)	49.5 (8.5)	43.9 (11.3)	45.1 (7.66)
Range	25–64	27–71	29–60
Fitzpatrick skin type, *n* (%)			
I		1 (3.1)	
II	1 (2.9)	20 (62.5)	1 (2)
III	8 (23.5)	8 (25.0)	14 (27.5)
IV	10 (29.4)	1 (3.1)	22 (43.1)
Not provided	15 (44.1)	2 (6.3)	14 (27.5)

#### Clinical photography

**Figure 1:**
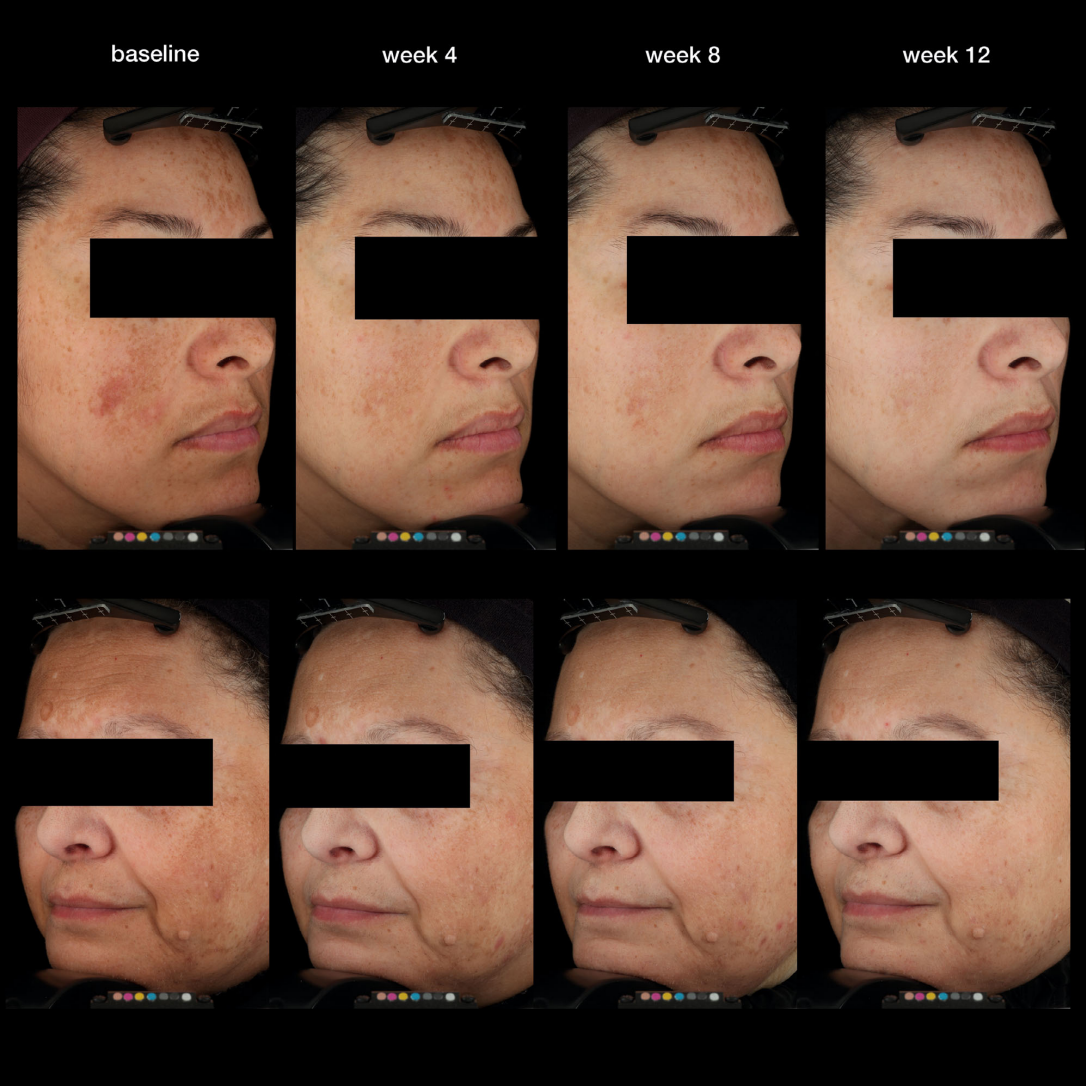
Selected digital images of two subjects before and after treatment with the combination (serum, day care SPF30) during the split‐face study.

Clinical photography demonstrated a visible improvement of the hyperpigmentation from baseline to Week 12 after treatment with the combination of serum and day care SPF30 (Fig. 1).

#### Clinical grading of skin condition

**Figure 2:**
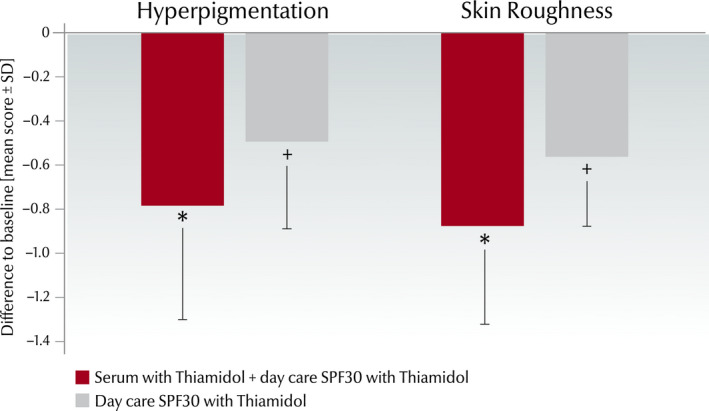
Clinical grading scores (0–4) from the split‐face study (*n* = 34) showing the difference (mean score ± SD) at week 12 versus baseline for hyperpigmentation and skin roughness. *Significant improvement (*P* < 0.001) compared to baseline and compared to treatment with only day care SPF30, ^+^significant improvement compared to baseline (*P* < 0.001).

After 12 weeks, application of the serum/day care SPF30 combination significantly improved both hyperpigmentation and skin roughness (−0.78 ± 0.52, −0.87 ± 0.45) versus baseline (*P* < 0.001) and versus the treatment with the day care SPF30 cream only (−0.49 ± 0.40; −0.56 ± 0.32) (*P* < 0.001; Fig. 2).

#### Self‐grading of skin condition

**Figure 3:**
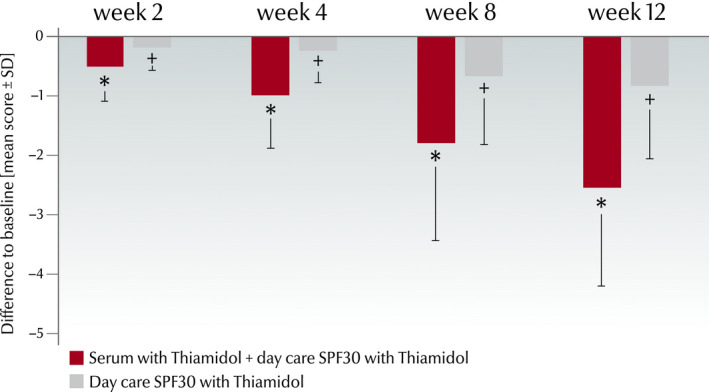
Self‐assessment of hyperpigmentation by modified Griffiths’ score (0–9) in the split‐face study. Differences of the mean hyperpigmentation scores (mean ± SD, *n* = 34) at each time point versus baseline. *Significant improvement (*P* < 0.001) compared with baseline and with treatment with day care SPF30 cream only, ^+^ Significant improvement compared with baseline (*P* < 0.001).

The self‐assessment also demonstrated at all‐time points statistically significant improvement of the mean hyperpigmentation scores of the face sides treated with the combination versus baseline (week 2: −0.50 ± 0.60; week 4: −0.99 ± 0.91; week 8: −1.79 ± 1.67; and week 12: −2.54 ± 1.70) and versus the sides treated with the day care SPF30 only (week 2: −0.18 ± 0.39; week 4: −0.25 ± 0.53; week 8: −0.68 ± 1.15; and week 12: −0.82 ± 1.26; *P* < 0.001) (Fig. 3).

#### hMASI

**Figure 4:**
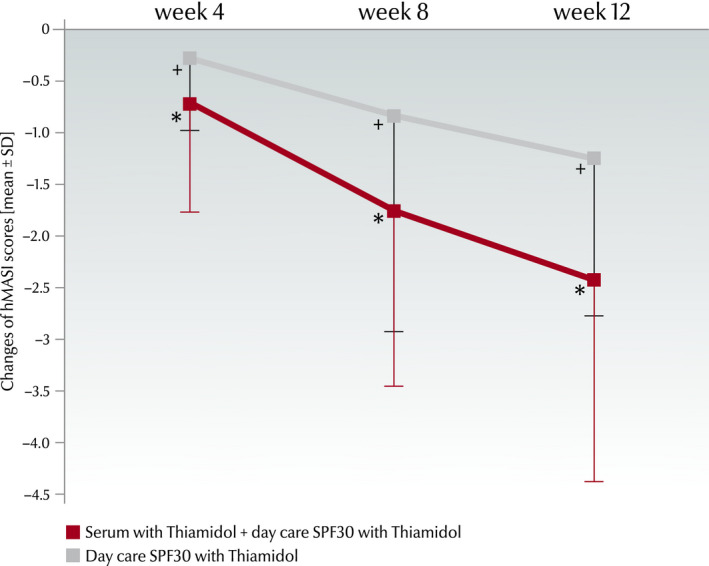
Changes of hMASI scores (mean ± SD) from baseline to week 12. Scores were assessed after 4, 8 and 12 weeks of treatment. *Significant improvement (*P* < 0.001) compared with baseline and with treatment with day care SPF30 cream only, ^+^ significant improvement compared with baseline (*P* < 0.001).

The combination treatment led to statistically significant hMASI reduction (*P* < 0.001) at all‐time points versus baseline (week 4: −0.72 ± 1.05; week 8: −1.76 ± 1.69; and week 12: −2.43 ± 1.96) and versus the sides treated with the day care only (week 4: −0.29 ± 0.69; week 8: −0.84 ± 1.45; and week 12; −1.26 ± 1.52; Fig. 4). The hMASI decreased from 6.12 at baseline to 3.69 at week 12 at the face sides treated with the combination and from 6.42 at baseline to 5.15 at week 12 at the sides treated with the day SPF30 cream only.

#### Tolerability

The analysis of the tolerability assessments showed no statistically significant changes from baseline for any parameter (erythema, oedema, dryness, burning, stinging or itching) at any time point for both sides of the face.

### Real‐world study

#### Demographic characteristics

Between August 2018 and April 2019, a total of 83 subjects (82 females) with a mean age of 44.7 ± 9.2 years (range 27–71) were enrolled. Demographics are summarized in Table 3.

#### Clinical photography

**Figure 5:**
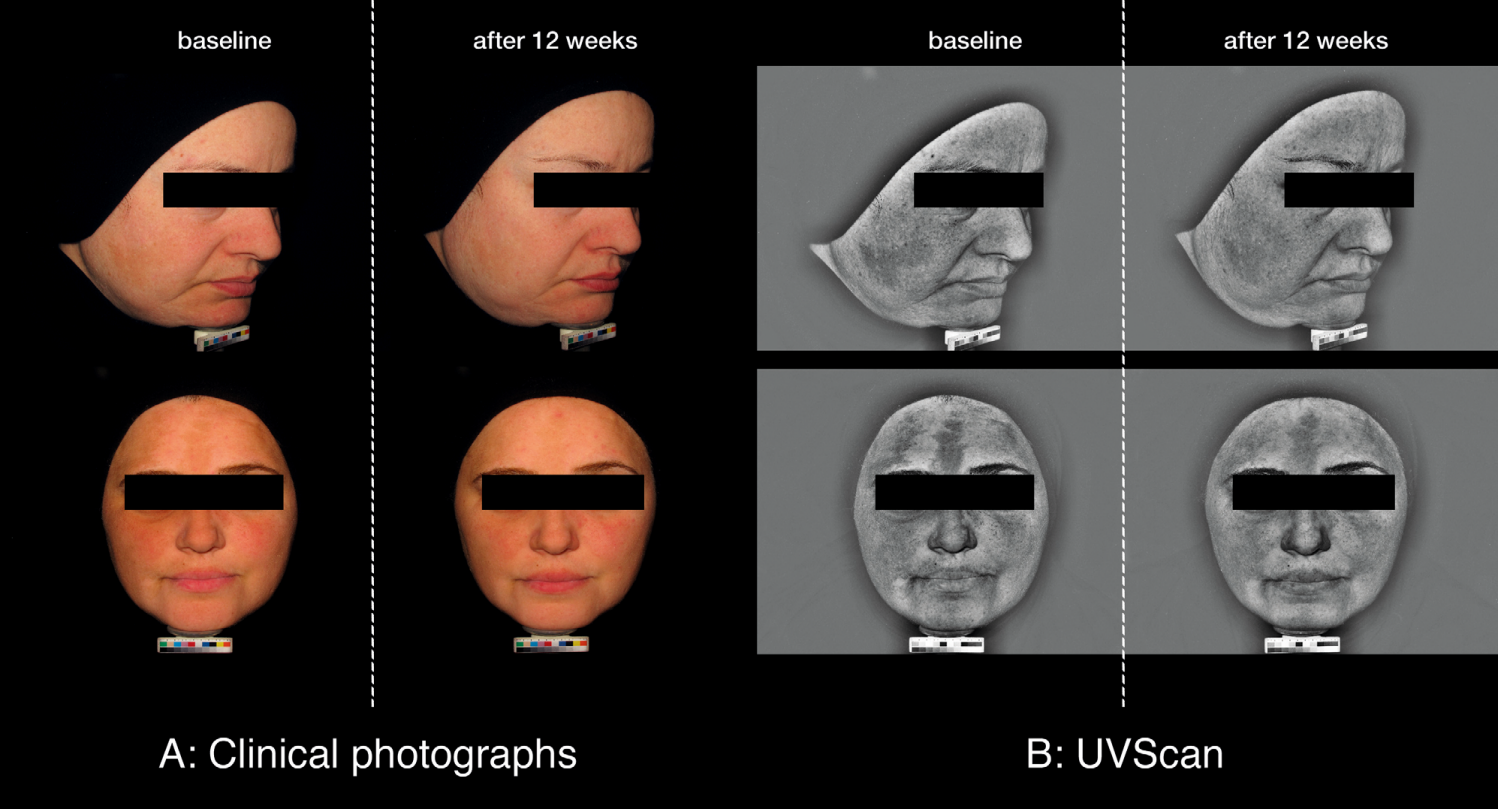
Representative Fotofinder Portrait^®^ base/UVSCAN^®^ images of a subject at baseline and after 12 weeks of treatment with the Thiamidol containing skin regimen (day care SPF30, serum and night care) during the real‐world study.

**Figure 6:**
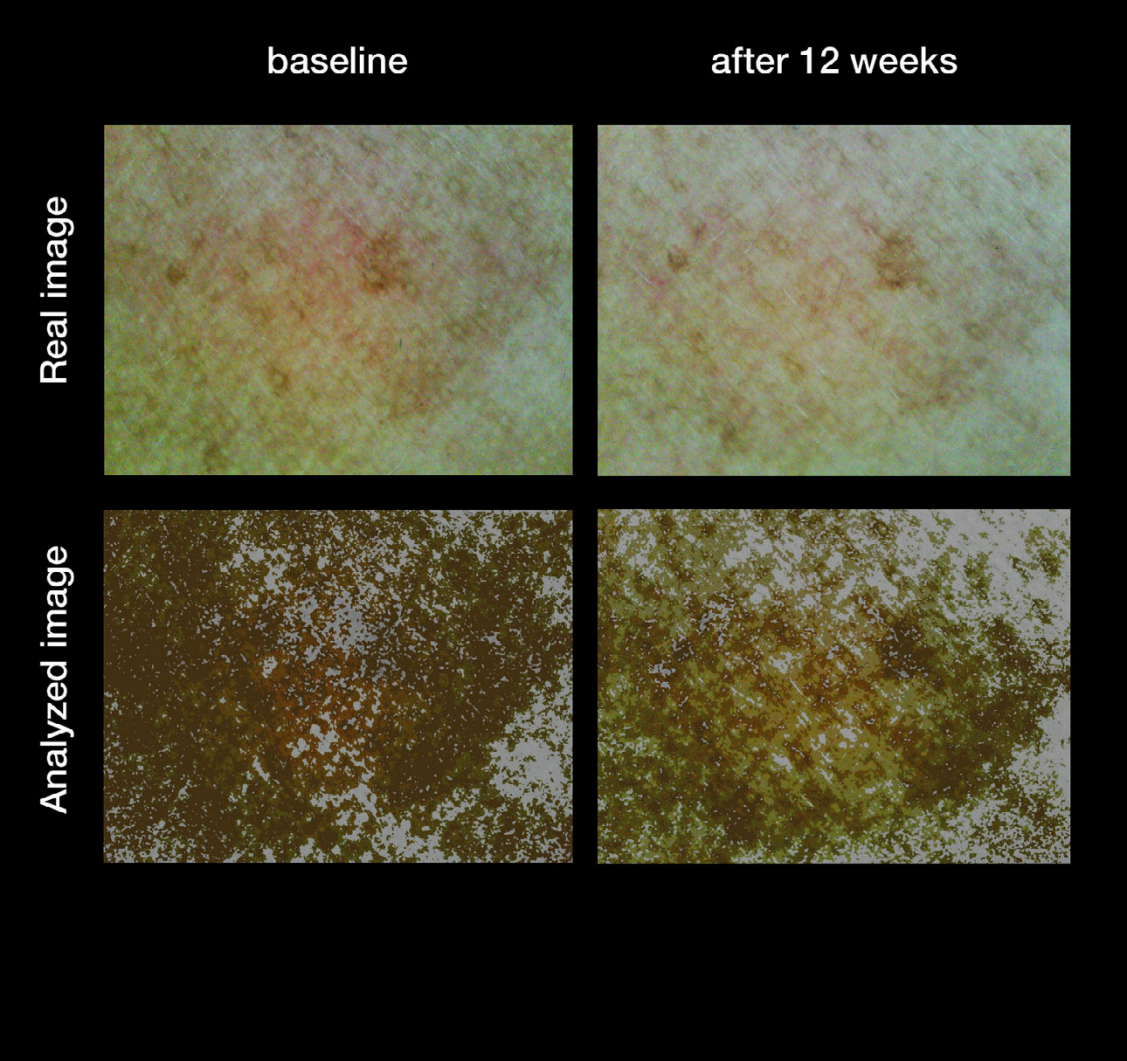
Representative Dermoprime^®^ images of a subject at baseline and after 12 weeks of treatment with the Thiamidol containing skin regimen (day care SPF30, serum and night care) during the real‐world study.

The improvement of hyperpigmentation after 12 weeks of treatment with the treatment regimen (day care SPF30, serum and night care) was documented by clinical photography (Figs 5 and 6).

#### Clinical grading of skin condition

**Figure 7:**
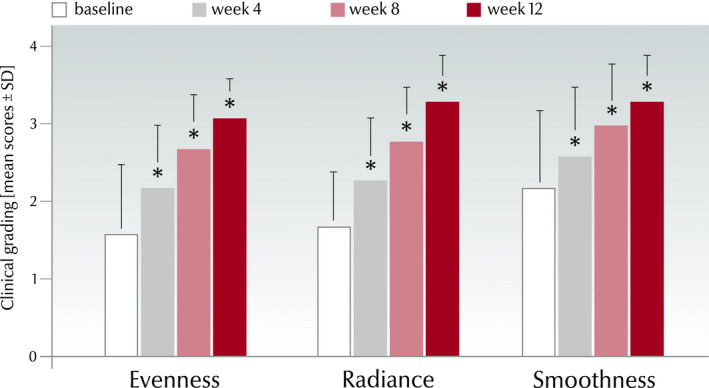
Clinical grading scores (0–4) at each time point for evenness, radiance and smoothness from all subjects (DE: Germany, AR: Argentina) considered for evaluation in the real‐world study (mean values ± SD, *n* = 83, *P < 0.001 vs. baseline and versus preceding visit).

In the course of the study, the means of all the skin condition parameters as assessed by the investigators significantly improved in comparison with baseline and with the previous visits (*P* < 0.001; Fig. 7). At week 12, mean evenness improved by 93.8% (from 1.6 ± 0.9 to 3.1 ± 0.5), radiance by 94.1% (from 1.7 ± 0.7 to 3.3 ± 0.6) and smoothness by 50.0% (from 2.2 ± 1.0 to 3.3 ± 0.6) versus baseline. From baseline to week 12, the evenness improved in 94% (*n* = 78) of the subjects and remained unchanged in 6.0% (*n* = 5), the radiance improved in 92.8% (*n* = 77) and remained unchanged in 7.2% (*n* = 6), and the smoothness improved in 78.3% (*n* = 65), remained unchanged in 18.1% (*n* = 15) and worsened in 3.6% (*n* = 3) of the subjects.

#### Self‐grading of skin condition

**Figure 8:**
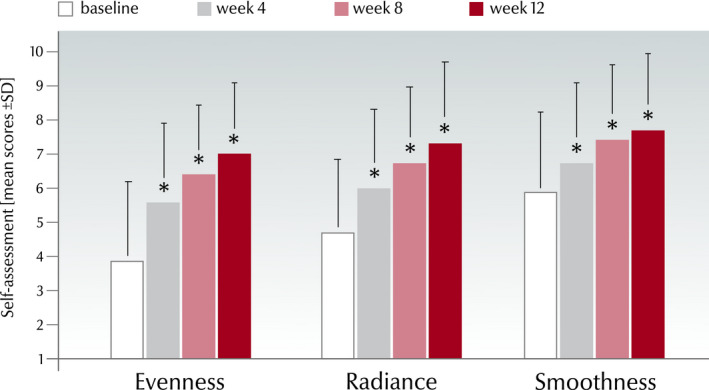
Self‐assessment by modified Griffiths’ score (1–10) at each time point for evenness, radiance and smoothness from all subjects considered for evaluation in the real‐world study (mean ± SD, *n* = 83, *P < 0.001 vs. baseline and versus preceding visit).

Corresponding to the clinical grading were also the results from the subjects’ self‐assessment (*P* < 0.001; Fig. 8). At week 12, mean evenness improved by 79.5% (from 3.9 ± 2.0 to 7 ± 1.6), radiance by 55.3% (from 4.7 ± 1.8 to 7.3 ± 1.9) and smoothness by 30.5% (from 5.9 ± 1.9 to 7.7 ± 1.7) versus baseline. Of the subjects, 89.2% (*n* = 74) found their evenness had improved, 9.6% (*n* = 8) that it was unchanged and 1.2% (*n* = 1) that it had worsened from baseline to week 12. The corresponding values for radiance were 86.7% (*n* = 72) improved, 6.0% (*n* = 5) unchanged and 7.2% (*n* = 6) worsened and for smoothness 77.1% (*n* = 64) improved, 15.7% (*n* = 13) unchanged and 7.2% (*n* = 6) worsened.

#### mMASI

**Figure 9:**
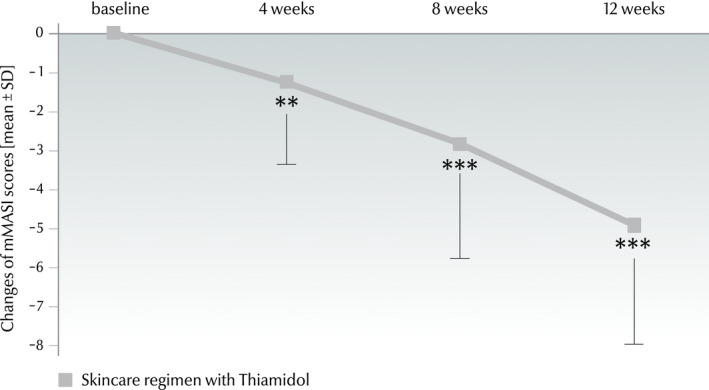
Changes of mMASI scores (mean ± SD) from baseline to week 4 (−1.3 ± 2.0), week 8 (−2.8 ± 2.9) and week 12 (−4.9 ± 3.0) (*n* = 32) in the real‐world study. Significances are indicated in comparison with baseline (**P ≤ 0.01, ***P ≤ 0.001).

The mMASI improved significantly from 8.5 ± 3.9 at baseline to 3.6 ± 2.6 at week 12 (*P* < 0.001). The improvement was statistically significant also when comparing visits 2‐4 to the corresponding preceding visits (*P* ≤ 0.001; Fig. 9).

#### Chromameter

**Figure 10:**
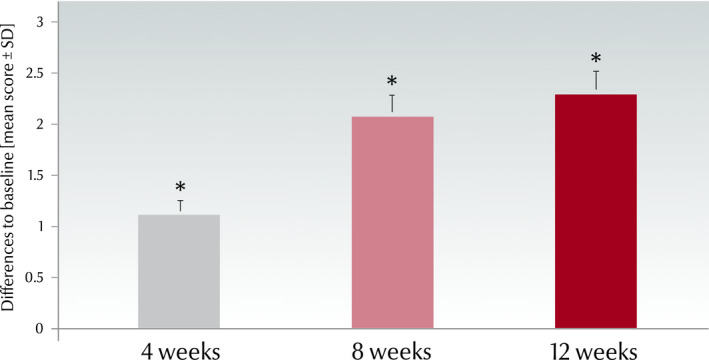
Change of chromameter luminosity (L* value, mean ± SD) versus baseline in the real‐world study (*n *= 30, *P < 0.001).

The mean luminosity value (L* parameter) increased significantly (*P* < 0.001) from 58.4 ± 3.5 at baseline to 59.6 ± 3.2 at week 4, to 60.5 ± 2.9 at week 8 and to 60.7 ± 2.8 at week 12, corresponding to an average change of 4.02%. The differences were significant between all possible pairs of L* means (*P* < 0.001) (Fig. 10).

#### Subjective assessment of product performance

Over 90% of the subjects rated all subjective performance questions positively, answering the respective questions with ‘yes’. The corresponding results are listed in Table 4.

**Table 4:** Self‐grading of product performance

Questions on product performance	Patients answering ‘yes’ (%)
The serum has a pleasant texture	97.6
The serum is skin‐compatible	97.6
The day care has a pleasant texture	97.5
The day care is skin‐compatible	91.4
The night care has a pleasant texture	92.8
The night care is skin‐compatible	92.6
The products are suitable for my skin	93.8
My skin feels cared for	93.8
My dark spots appear less pronounced	97.6
The products improve the appearance of dark spots	95.2
The products even out my skin	91.6
My skin tone looks more even	93.8
The products provide a smooth skin	90.0
The products provide a radiant complexion	93.7

#### Tolerability

The investigators rated the tolerability of the product as very good in 73.5% (*n* = 61), as good in 22.9% (*n* = 19) and as satisfactory in 3.6% (*n* = 3) of the subjects.

## Discussion

The split‐face study demonstrated in the clinical setting and already after two weeks that four‐times daily Thiamidol was visibly and significantly more effective than twice‐daily in all assessments and corresponding time points. The release of the active from the two products (serum and day care) is expected to be comparable. Therefore, the results from the split‐face study are indicating the benefit of a higher application frequency over a lower. The significant improvement continued until the end of the study in both sides of the face. The second study showed the efficacy, day‐to‐day acceptability and excellent tolerability, of a typical face care regimen containing the human tyrosinase inhibitor in a real‐world setting. These results underline the real‐life suitability of the products and their compatibility with the usual make‐up and cleansing routines.

The reduction of the MASI Score was in both studies significant, considerable, and in‐line with earlier made observations [20]. The value of hMASI in the split‐face study was smaller because, per definition, it represents the area of half the face. Duplication of the hMASI corresponds to mMASI in the real‐world study. We chose the modified MASI because it was shown to be a reliable, valid and responsive to change method for the assessment of melasma severity. Besides, mMASI was demonstrated to be more convenient to use than MASI [25].

In addition to hyperpigmentation also skin roughness, evenness, radiance and smoothness improved significantly. Investigators and subjects confirmed the visible improvements, which were also documented by clinical photography and chromametry. The two latter standardized methods are objective, excluding interobserver variability and difficulty of remembering the baseline condition at the subsequent visit [24]. However, subjective assessments of efficacy are also essential to estimate compliance, especially for conditions such as hyperpigmentation, the treatment of which is challenging and frustrating for patients and physicians [5, 10].

In the real‐world study, the overall skin improvements and tolerability of the three‐product regimen led to good overall acceptance. Over 90% of the subjects agreed that the products have a pleasant texture and are skin‐compatible. Results, which are supporting adherence to the treatment. Therefore, we conclude the suitability of this three‐product regimen for day‐to‐day life.

The studies were conducted on both sides of the hemisphere, covered most seasons except summer and various sun intensities to reflect real‐world conditions. Based on an earlier study [20], in which half of the patients applied the treatment during the low ambient‐UV time of the year and the other half during the high ambient‐UV period, we expected no seasonal effects. Nevertheless, as UV‐radiation can promote or worsen hyperpigmentation [28, 29], sunscreen use is mandatory [30, 31]. Therefore, SPF 30 was added in the day care product, which was used by all subjects, excluding thus possible confounding by sun exposure. Because subjects were not allowed to use other face care products for the duration of the studies, we can assume that no other substances influenced the results. Furthermore, a safety assessment including photosensitivity tests demonstrated that Thiamidol does not induce UV sensitivity (data on file).

As hyperpigmentation may have intrinsic or extrinsic triggers [32] affecting all ages, we included adult subjects of 25–65 years in the two studies. The studies’ populations were too small for age‐specific sub‐group analyses, something to consider for the future. However, the lightening effect of Thiamidol on age spots was shown earlier [17]. Similarly, previous studies (and yet unpublished results) showed that the molecule is efficacious in all ethnicities [20]. Thus, in the two studies reported here, we did not focus on ethnic groups. Nevertheless, as hyperpigmentation has a higher prevalence in darker skin types [33], a future study could focus on those. Furthermore, because of the higher prevalence, we focused here on women. Only one man was included in the real‐world study, not allowing sex‐specific conclusions.

The results reported here supplement and confirm the so far with Thiamidol conducted investigations. Unfortunately, it was not possible to determine all parameters in every centre, affecting the comparability of the results. However, the overall design, duration, visit frequency and methods chosen here are typical for this kind of studies [34, 35, 36].

In conclusion, Thiamidol four‐times daily is well tolerated and provides significant visible improvement of hyperpigmentation versus baseline and twice‐daily application. The regimen approach with the three products (serum, day care SPF30 and night care) is also well tolerated, effective and suitable for the day‐to‐day life of people suffering from facial hyperpigmentation.
